# IL-5 and GM-CSF, but Not IL-3, Promote the Proliferative Properties of Inflammatory-like and Lung Resident-like Eosinophils in the Blood of Asthma Patients

**DOI:** 10.3390/cells11233804

**Published:** 2022-11-28

**Authors:** Jolita Palacionyte, Andrius Januskevicius, Egle Vasyle, Airidas Rimkunas, Ieva Bajoriuniene, Skaidrius Miliauskas, Kestutis Malakauskas

**Affiliations:** 1 Department of Pulmonology, Lithuanian University of Health Sciences, LT-44307 Kaunas, Lithuania; 2 Laboratory of Pulmonology, Department of Pulmonology, Lithuanian University of Health Sciences, LT-44307 Kaunas, Lithuania; 3 Department of Immunology and Allergology, Lithuanian University of Health Sciences, LT-44307 Kaunas, Lithuania

**Keywords:** asthma, eosinophil, IL-3, IL-5, GM-CSF, proliferation, airway smooth muscle cells, gene expression

## Abstract

Blood eosinophils can be described as inflammatory-like (iEOS-like) and lung-resident-like (rEOS-like) eosinophils. This study is based on the hypothesis that eosinophilopoetins such as interleukin (IL)-3 and IL-5 and granulocyte-macrophage colony-stimulating factor (GM-CSF) alter the proliferative properties of eosinophil subtypes and may be associated with the expression of their receptors on eosinophils. We investigated 8 individuals with severe nonallergic eosinophilic asthma (SNEA), 17 nonsevere allergic asthma (AA), and 11 healthy subjects (HS). For AA patients, a bronchial allergen challenge with *Dermatophagoides pteronyssinus* was performed. Eosinophils were isolated from peripheral blood using high-density centrifugation and magnetic separation methods. The subtyping of eosinophils was based on magnetic bead-conjugated antibodies against L-selectin. Preactivation by eosinophilopoetins was performed by incubating eosinophil subtypes with IL-3, IL-5, and GM-CSF, and individual combined cell cultures were prepared with airway smooth muscle (ASM) cells. ASM cell proliferation was assessed using an Alamar blue assay. The gene expression of eosinophilopoetin receptors was analyzed with a qPCR. IL-5 and GM-CSF significantly enhanced the proliferative properties of iEOS-like and rEOS-like cells on ASM cells in both SNEA and AA groups compared with eosinophils not activated by cytokines (*p* < 0.05). Moreover, rEOS-like cells demonstrated a higher gene expression of the IL-3 and IL-5 receptors compared with iEOS-like cells in the SNEA and AA groups (*p* < 0.05). In conclusion: IL-5 and GM-CSF promote the proliferative properties of iEOS-like and rEOS-like eosinophils; however, the effect of only IL-5 may be related to the expression of its receptors in asthma patients.

## 1. Introduction

Eosinophils are terminally differentiated, bone-marrow-derived, granule-containing leukocytes [1]. Typically, these cells are present in relatively low numbers in peripheral blood, about one percent of all leukocytes [2]. Despite the low number, they are considered the most important inflammatory cells in asthma. Eosinophils have primarily been regarded as proinflammatory cells involved in host protection against parasite infection and the immunopathology of allergic diseases. It is now known that eosinophils contribute to a wide variety of physiological and pathological processes, including metabolism, tissue remodeling and development, epithelial regulation, and immunoregulation, indicating that these cells may play a crucial role in metabolic regulation and organ function and diseases [3,4].

Nearly half of asthma patients have predominant eosinophilic inflammation in their blood and airways [5]. A higher eosinophil count in the airway is associated with bronchial hyperresponsiveness, airway inflammation, and airflow limitation. It is determinative of intense asthma symptoms, more frequent exacerbations, and a more severe disease course [4,6]. At least two subtypes of eosinophils have been identified and differ according to their role in asthma pathogenesis. One subtype is the lung-resident eosinophil (rEOS), and the other is the inflammatory eosinophil (iEOS) [5]. Eosinophil count in the blood or lungs, survival, maturation, and activation are promoted by cytokines called eosinophilopoetins, of which interleukin (IL) 3, IL-5, and granulocyte-macrophage colony-stimulating factor (GM-CSF) are examples. Eosinophilopoetins are also observed to affect eosinophil proliferative properties [7] and the hypertrophy and proliferation of pulmonary structural cells, including airway smooth muscle (ASM) cells [8].

Eosinophils express receptors for multiple growth factors and cytokines on their surface [9]. Eosinophilopoetins may enhance the receptor expression of IL-3, IL-5, and GM-CSF, which can lead to excessive signal transduction and alter the disease course [10]. IL-3, IL-5, and GM-CSF are classified as β-chain cytokines because they share a common β-chain and have unique cytokine-specific α-chains [11,12,13]. Eosinophilopoetins possess unique characteristics for eosinophil activation; that way, different cytokines can differentially affect eosinophil functions, and eosinophil subtypes can exhibit different reactions to the same eosinophilopoetin [14]. The effects of eosinophilopoetins depend not only on the concentration of these cytokines but also on the number of receptors on the eosinophil surface [13]. For this reason, anticytokine receptor antibodies, in addition to anticytokines, may also be considered as an effective therapy for patients with asthma who are not responding to conventional treatment [15].

Eosinophils contain about 200 morphologically distinct cytoplasmic granules containing over 35 types of mediators [16]. They produce cytokines, chemokines, and growth factors that cause various structural changes in the airways in a process referred to as airway remodeling [17]. Airway remodeling is characterized by an increased ASM mass due to hyperplasia and hypertrophy, subepithelial fibrosis, goblet cell hyperplasia, submucosal mucus gland hypertrophy, and other pathological processes [18]. One of the main pathological processes that causes the clinical manifestation of asthma and severity of the disease is hyperplasia of ASM cells [9]. ASM cells usually maintain their airway diameter by modulating airway tone, so the dysfunction of ASM cells is an important component of obstructive pulmonary diseases, particularly asthma. External stimuli such as allergens, dust, air pollutants, and changes in environmental temperature provoke ASM cell hypertrophy and proliferation and cause ASM cell dysfunction [19].

Most patients achieve adequate symptom control through inhaled glucocorticoid-based treatment. However, a proportion of patients continue to experience persistent and severe disease despite good adherence to inhaled steroids, leading to frequent exacerbations requiring treatment with oral glucocorticoids. Regardless of the success of biologic anti-IL-5 therapy in severe eosinophilic asthma, a subset of patients continue to have uncontrolled symptoms and exacerbations [4]. It is therefore appropriate to continue to delve into the mechanisms of eosinophilic asthma to improve asthma treatment. The novelty of our study is that we activate blood eosinophil subtypes with eosinophilopoetins and use them in combined cultures with ASM cells in an attempt to demonstrate their different proliferative properties. We also hypothesize that the bronchial allergen challenge might enhance the response of certain eosinophil subtypes to activation by eosinophilopoetins. In this research, therefore, we aimed to investigate the effect of IL-3, IL-5, and GM-CSF on the proliferative properties of blood rEOS-like and iEOS-like cells that might be associated with the expression of their receptors in asthma patients.

## 2. Materials and Methods

### 2.1. Investigation Protocol

All subjects were introduced to the study and allowed sufficient time to familiarize themselves with the study protocol, and all questions were answered. The investigations were started after the person signed the investigation protocol, which was approved for use on humans by the regional biomedical ethics committee (BE-2-58). All data were anonymized by assigning unique numbers. The study was registered on ClinicalTrial.gov with the identification number NCT04542902.

### 2.2. Study Design

This study included 46 adults. Men and women aged 18–80 participated in the study. The subjects were divided into three groups: healthy subjects (HS), SNEA, and AA patients. Patients were recruited from the Department of Pulmonology at the Hospital of Lithuanian University of Health Sciences Kaunas Clinics. The inclusion and exclusion criteria are shown in Table 1.

All participants were invited to the study no later than four weeks after the approval of the inclusion or exclusion criteria. Participants were required to stay 30 min to 2 h from their arrival at the hospital. AA patients visited the hospital twice (at baseline and 24 h after the bronchial allergen challenge) and all other participants visited the hospital once. Before beginning the research, an informed consent form was given to each research subject, and research started after this was signed by the subject. The research plan is shown in Table 2.

**Figure 1:**
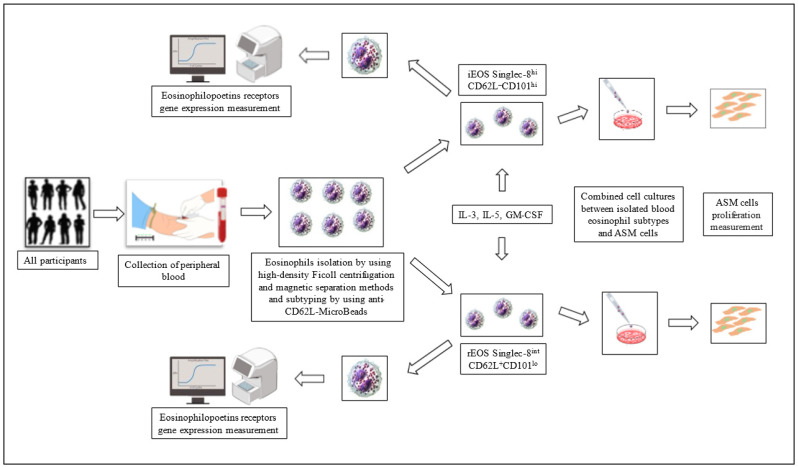
Graphical overview of the study design. ASM—airway smooth muscle; IL—interleukin; GM-CSF—granulocyte-macrophage colony-stimulating factor; CD—cluster of differentiation.

A graphic of study design is shown in Figure 1.

### 2.3. Complete Blood Count and Immunoglobulin E

Peripheral blood samples of participants were drawn into vacutainers containing dipotassium ethylenediaminetetraacetic acid (K2EDTA) (BD Vacutainer^®^, Becton Dickinson UK Ltd., Wokingham, UK). For all routine clinical chemistry assays, samples were directly transported to the laboratory of our hospital. XE-5000 (Sysmex, Kobe, Japan) and UniCel^®^ DxH 800 Coulter^®^ Cellular Analysis System automated hematology analyzer (Beckman Coulter, Miami, FL, USA, JAV) was used for the complete blood count test. An AIA-2000 automated immunoassay analyzer (Tosoh Bioscience, South San Francisco, CA, USA, JAV) was used for the immunoglobulin E (Ig E) level test. Blood tests were processed under tightly controlled and monitored conditions. A detailed blood analysis is described below.

### 2.4. Spirometry

Spirometry was performed on all participants at least three times. An ultrasonic spirometer was used to test the lung function (Ganshorn Medizin Electronic, Niederlauer, Germany). Details of the techniques for performing this procedure are described in [20].

### 2.5. Metacholine Challenge Test

A metacholine challenge test was performed on AA patients, except those with airflow limitations noted at the screening visit. For the inhalation of methacholine, a dosimeter was used (ProvoX, Ganshorn Medizin Electronic, Niederlauer, Germany), and lung function was checked every 2 min. The methacholine challenge test was completed when the forsed expiratory volume in 1 s (FEV_1_) decreased by 20%. Details of the techniques for performing this procedure are described in [20].

### 2.6. Bronchial Reversibility Test

A bronchial reversibility test was performed only for 5 subjects in the AA group that had airflow limitations noted at the screening visit. This test was performed using a Ganshorn spirometer (Ganshorn Medizin Electronic, Niederlauer, Germany). Spirometry was performed before and 15 min after salbutamol inhalation (400 mcg). The procedure performance technique is described in [21].

### 2.7. Skin Prick Testing

All patients underwent skin prick allergy testing with *D. pteronyssinus, D. farinae*, cat and dog dander, five mixed grass pollens, birch pollen, mugwort, Alternaria, Aspergillus, and Cladosporium using standardized allergen extracts (Stallergenes, S.A., Antony, France). Details of the techniques for performing this procedure are described in [20].

### 2.8. Fractional Exhaled Nitric Oxide Test

A fractional exhaled nitric oxide (Fe_NO_) analysis was performed on all study participants. For subjects of the AA group, Fe_NO_ levels were recorded twice: before and 24 h after the bronchial challenge with the *D. pteronyssinus* allergen. We used a single exhalation and electrochemical assay (NIOX VERO, Circassia, UK) according to the methodology described in [20].

### 2.9. Bronchial Challenge with D. pteronyssinus

A bronchial challenge with the *D. pteronyssinus* allergen (DIATER, Madrid, Spain) was performed on all AA group participants who were sensitized to *D. pteronyssinus*. For the inhalation of this allergen, we used a dosimeter (ProvoX, Ganshorn Medizin Electronic, Niederlauer, Germany). The bronchial challenge with the *D. pteronyssinus* test was stopped when the FEV_1_ decreased by 20%. The whole procedure is described in [20].

### 2.10. Peripheral Blood Cell Analysis

Approximately 40 mL of peripheral blood from each subject was collected into sterile vacutainer tubes with ethylenediaminetetraacetic acid (EDTA) and delivered to the laboratory. In group AA patients, blood tests were repeated and tested 24 h after the bronchial challenge with *D. pteronyssinus*. Erythrocytes and granulocytes were isolated using high-density Ficoll centrifugation. Eosinophils were separated using magnetic separation methods. The phenotyping of eosinophils was based on magnetic bead-conjugated antibodies against L-selectin. These procedures are described in detail in [22].

### 2.11. Eosinophil Activation

Isolated eosinophil subtypes were transferred into separate Eppendorf^®^ vacutainer tubes with different eosinophilopoetins: IL-3, IL-5, or GM-CSF (all three eosinophilopoetins were used at 10 ng/mL). We prepared 6 vacutainers and adjusted the volume to 500 µL using a DMEM cell culture. The samples were incubated in a thermostat for 3 h and then centrifugated for 10 min (speed 400 g). After centrifugation, the supernatant was decanted into a separate container. The eosinophils remaining in the tube were resuspended with the DMEM cell culture and were poured onto ASM cells.

### 2.12. Airway Smooth Muscle Cell Proliferation Assay

Healthy human ASM cells were isolated from one donor. For each study, individual combined cell cultures between isolated and affected by eosinophilopoetins blood eosinophil subtypes and ASM cells were prepared. We used 0.5 × 10^5^ viable eosinophils and 2 × 10^5^ ASM cells grown for 3 days for all experiments. ASM cell proliferation was assessed using an Alamar blue assay, with details described in [22].

### 2.13. Gene Expression Assessment

The expression levels of IL-3, IL-5, and GM-CSF genes were determined for both eosinophil subtypes with qPCR using the commercial Power SYBR^®^ Green RNA-to-CT™ 1-Step kit (Applied Biosystems, Foster City, CA, USA). The process is described in detail [22]. The gene expression changes were evaluated based on a fold change between different eosinophil subtypes in the same group and of the same eosinophil subtypes between different groups.

### 2.14. Statistical Analysis

A statistical analysis was performed using SPSS statistical software (IBM SPSS Statistics 20; Chicago, IL, USA). The Shapiro–Wilk test was used to test the assumption of normality in the data distribution. The data distribution did not pass the normality test, so a nonparametric Mann–Whitney two-sided U-test and Wilcoxon matched-pair, signed-rank, two-sided test were used. The minimal limit for a statistically significant difference in values was *p* < 0.05.

## 3. Results

### 3.1. Study Subject Characteristics

In this study, 18 SNEA patients, 17 steroid-free nonsevere AA patients, and 11 HS (a total of 46 adults) were included. Demographic and clinical characteristics of the study population are shown in Table 3. The SNEA patients were significantly older than the AA and HS groups, as SNEA usually manifests as a late-onset disease. A higher body mass index value was recorded for SNEA than for AA and HS. A significant decrease in FEV_1_ was observed only in SNEA patients. The blood eosinophil counts were higher in SNEA and AA patients than HS patients, with a higher count in the SNEA group. No differences in Fe_NO_ levels were found in both asthma groups. The IgE levels in the serum were higher in SNEA and AA patients than HS patients, with the highest level in the AA group.

### 3.2. Effect of IL-3, IL-5, GM-CSF on Eosinophil Subtypes Pro-Proliferative Properties

IL-3 did not affect the proliferative properties of any eosinophil subtypes in the SNEA group (*p* > 0.05); however, IL-5 and GM-CSF significantly enhanced the effect on the proliferative properties of iEOS-like and rEOS-like cells (*p* < 0.05). Compared with ASM cells incubated with nonactivated eosinophils, the ASM cell number increased by 47.6 ± 6.5% and 51.2 ± 7.3% after a 72 h coculture with iEOS-like cells and by 48.0 ± 5.8% and 62.4 ± 4.5% after a 72 h coculture with rEOS-like for activation with IL-5 and GM-CSF, respectively (*p* < 0.05). Compared with the SNEA group, there was no significant difference (*p* > 0.05) in ASM cell number for coculture with IL-3-, IL-5-, and GM-CSF-activated iEOS-like cells.

In the AA group, IL-5 and GM-CSF significantly enhanced the effect of iEOS-like and rEOS-like cells on ASM cell proliferation (*p* < 0.05). Compared with ASM cells incubated with nonactivated eosinophils, the ASM cell number increased by 26.6 ± 4.1% and 34.8 ± 4.3% after a 72 h coculture with iEOS-like cells and 38.3 ± 1.6% and 35.1 ± 3.7% for rEOS-like cells (*p* < 0.05) when activated with IL-5 and GM-CSF, respectively. At the same time, IL-3 had no significant impact on any eosinophil subtypes (*p* > 0.05). Compared with the AA group, there was no significant difference (*p* > 0.05) in ASM cell number for the coculture with IL-3-, IL-5-, and GM-CSF-activated iEOS-like cells (*p* > 0.05).

In the HS group, IL-5 had a significant effect on the proliferative properties of iEOS-like cells (*p* < 0.05). The ASM cell number after 72 h of incubation in a coculture with IL-5 activated iEOS-like cells increased by 18.6 ± 2.9% compared with ASM cells incubated with nonactivated eosinophils (*p* < 0.05). At the same time, IL-3 and GM-CSF had no significant impact on any eosinophil subtypes (*p* > 0.05). Compared with the HS group, there was no significant difference (*p* > 0.05) in ASM cell number for the coculture with IL-3-, IL-5-, and GM-CSF-activated iEOS-like cells (*p* > 0.05).

The effect of IL-5 on the proliferative properties of iEOS-like cells was higher in the SNEA group than in the AA group (ASM cell number increased by 47.6 ± 6.5% vs. 26.6 ± 4.1%). Meanwhile, the effect of GM-CSF on the proliferative properties of rEOS-like cells was higher compared with the AA group (ASM cell number increased by 62.4 ± 4.5% vs. 35.1 ± 3.7%, *p* < 0.05).

**Figure 2:**
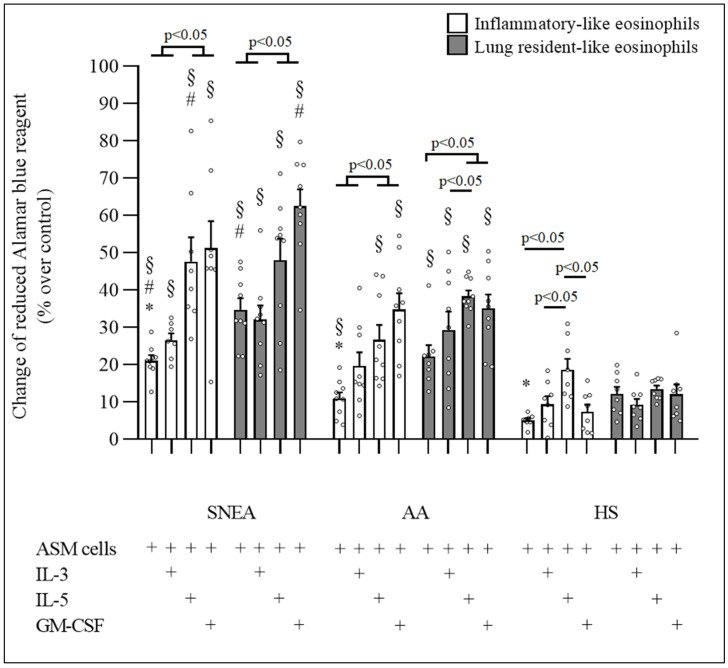
Effect of eosinophilopoetins on proliferative properties of blood eosinophil subtypes. ASM—airway smooth muscle; IL—interleukin; GM-CSF—granulocyte-macrophage colony-stimulating factor. Severe nonallergic eosinophilic asthma (SNEA) group n = 10, nonsevere allergic asthma (AA) group n = 9, healthy subjects (HS) group n = 8. Data presented as the mean ± standard error of the mean. * *p* < 0.05 compared with appropriate rEOS-like cells in the same group. ^#^ *p* < 0.05 compared with appropriate eosinophil subtypes from AA group. ^§^ *p* < 0.005 compared with appropriate eosinophil subtypes from HS group. Statistical analysis: between investigated groups, Mann–Whitney two-sided U-test; within one study group, Wilcoxon matched-pair signed-rank two-sided test. Data presented as the mean ± standard error of the mean.

The effect of IL-3 and GM-CSF on the proliferative properties of iEOS-like cells was higher in the AA group than the HS group (ASM cell number increased, respectively, by 19.6 ± 3.6%; 34.8 ± 4.3% vs. 9.4 ± 2.1%; 7.4 ± 1.9%, *p* < 0.05) while the effect of IL-3, IL-5, and GM-CSF on the proliferative properties of rEOS-like cells was higher in the AA group than the HS group (ASM cell number increased by 29.3 ± 4.9%, 38.3 ± 1.6%, 35.1 ± 3.7% vs. 9.3 ± 1.6%, 13.4 ± 1.0%, 12.0 ± 2.7%, respectively, *p* < 0.05). The effect of IL-3, IL-5, and GM-CSF on the proliferative properties of both eosinophil subtypes was higher in the SNEA group than the HS group (ASM cell number increased by 26.6 ± 1.8%, 47.6 ± 6.5%, and 51.2 ± 7.2%, respectively, for iEOS-like cells and 32.1 ± 3.8%, 48.0 ± 5.8%, and 62.4 ± 4.5%, respectively, for rEOS-like cells vs. 9.4 ± 2.1%, 18.6 ± 2.9%, and 7.4 ± 1.9%, respectively, for iEOS-like cells and 9.3 ± 1.6%, 13.4 ± 1.0%, and 12.0 ± 2.7%, respectively, for rEOS-like cells, *p* < 0.05, Figure 2).

### 3.3. Effect of Eosinophilopoetins on Pro-Proliferative Properties of Eosinophil Subtypes after Allergen Activation In Vivo

The effect on the proliferative properties of eosinophil subtypes following an in vivo provoked acute allergic asthma episode after the bronchial allergen challenge was determined by comparing the results before and 24 h after the allergen challenge of the same subject.

The bronchial allergen challenge significantly increased the effect of IL-5 and GM-CSF on the proliferative properties of rEOS-like cells (*p* < 0.05). After 72 h of incubation in coculture with IL-5- and GM-CSF-activated rEOS-like cells, the ASM cell number increased by 61.1 ± 7.0% and 66.2 ± 7.3%, respectively, compared with the ASM cells incubated with nonactivated eosinophils, and by 38.3 ± 1.6% and 35.1 ± 3.7% (*p* < 0.05), respectively, compared with ASM cells incubated with IL-5- and GM-CSF-activated eosinophils before bronchial allergen challenge. Meanwhile, IL-3 had no significant effect on rEOS-like cells (*p* > 0.05).

**Figure 3:**
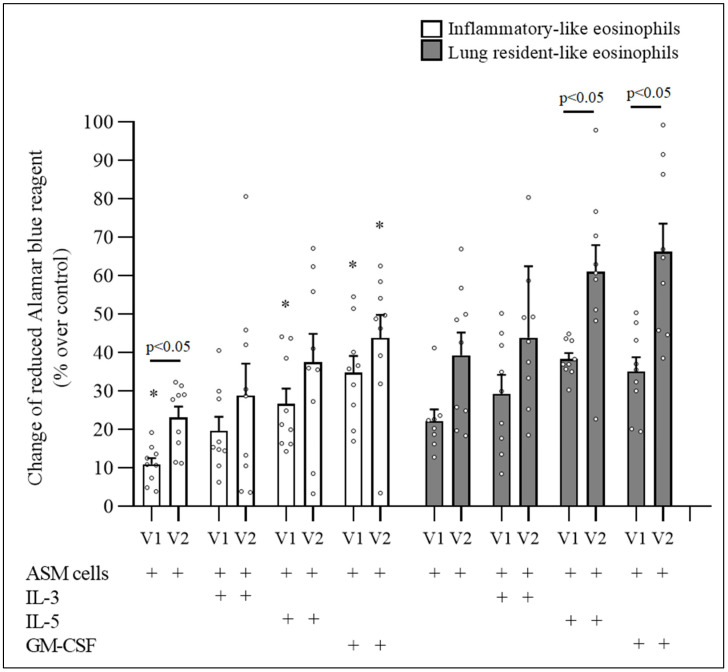
The effect of bronchial allergen challenge on proliferative properties of blood eosinophil subtypes in allergic asthma. ASM—airway smooth muscle; IL—interleukin; GM-CSF—granulocyte-macrophage colony-stimulating factor, V1—visit 1 (before bronchial allergen challenge); V2—visit 2 (24 h after bronchial allergen challenge). Results from independent experiments of AA, n = 8. Data presented as the mean ± standard error of the mean. * *p* < 0.05 compared with appropriate rEOS-like cells. Statistical analysis: between investigated groups, Mann–Whitney two-sided U-test; within one study group, Wilcoxon matched-pair signed-rank two-sided test. Data presented as the mean ± standard error of the mean.

The bronchial allergen challenge did not significantly change the ASM cell number after 72 h of incubation in coculture with IL-3-, IL-5-, and GM-CSF-activated iEOS-like cells compared with the same cells before the bronchial allergen challenge (*p* > 0.05) (Figure 3).

### 3.4. Gene Expression of IL-3R, IL-5R, and GM-CSFR

**Figure 4:**
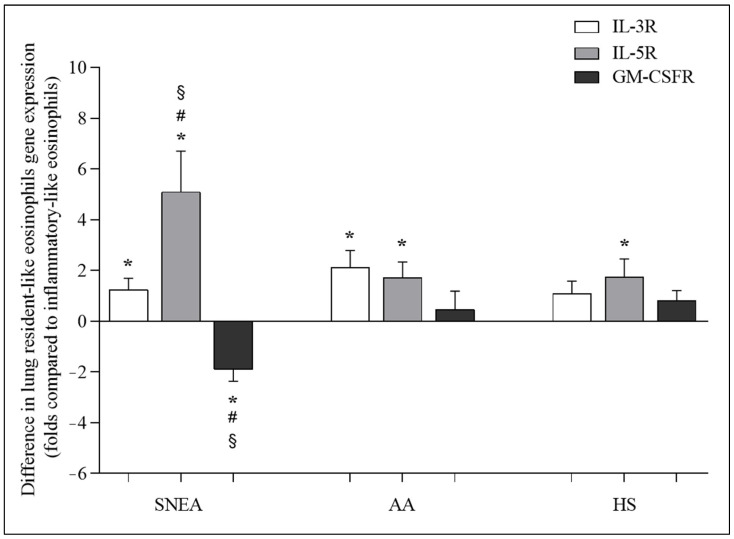
Differences in expression of IL-3R, IL-5R, and GM-CSFR genes between lung resident-like and inflammatory-like eosinophils in the same groups. IL-3R—interleukin 3 receptor; IL-5R—interleukin 5 receptor; GM-CSFR—granulocyte-macrophage colony-stimulating factor receptor. Severe nonallergic eosinophilic asthma (SNEA) group n = 10, nonsevere allergic asthma (AA) group n = 8, healthy subjects (HS) group n = 11. Data presented as the mean ± standard error of the mean, fold change over expression of IL-3R, IL-5R, and GM-CSFR gene on inflammatory-like eosinophils. * *p* < 0.05 compared with inflammatory-like eosinophils; ^#^ *p* < 0.05 compared with AA group; ^§^ *p* < 0.05 compared with HS group. Statistical analysis: between investigated groups, Mann–Whitney two-sided U-test; within one study group, Wilcoxon matched-pair signed-rank two-sided test. Data presented as the mean ± standard error of the mean.

Compared with iEOS-like cells, rEOS-like cells isolated from the SNEA and AA groups showed a higher expression of the IL-3 receptor gene (respectively 1.2 ± 0.5; 2.1 ± 0.7 folds, *p* < 0.05). Meanwhile, in the HS group, the expression of the IL-3 receptor gene did not significantly differ (*p* > 0.05) between eosinophil subtypes. rEOS-like cells isolated from the SNEA, AA, and HS groups showed a higher expression of the IL-5 receptor gene compared with iEOS-like cells (5.1 ± 1.7-, 1.7 ± 0.7-, and 1.7 ± 0.8-fold, respectively, *p* < 0.05). In the SNEA group, a higher expression of the GM-CSF receptor gene was observed in the isolated iEOS-like compared with rEOS-like cells (1.9 ± 0.5-fold, *p* < 0.05). Meanwhile, in the AA and HS groups, the expression of the GM-CSF receptor gene did not significantly differ (*p* > 0.05) between the eosinophil subtypes (Figure 4).

**Figure 5:**
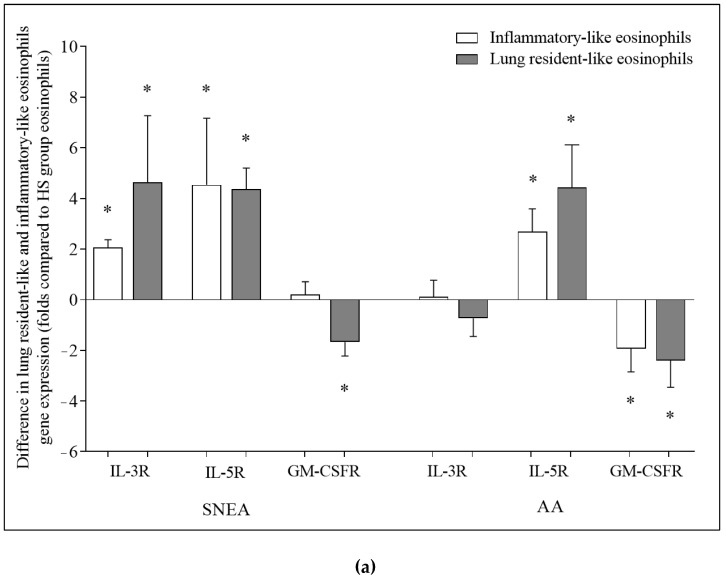

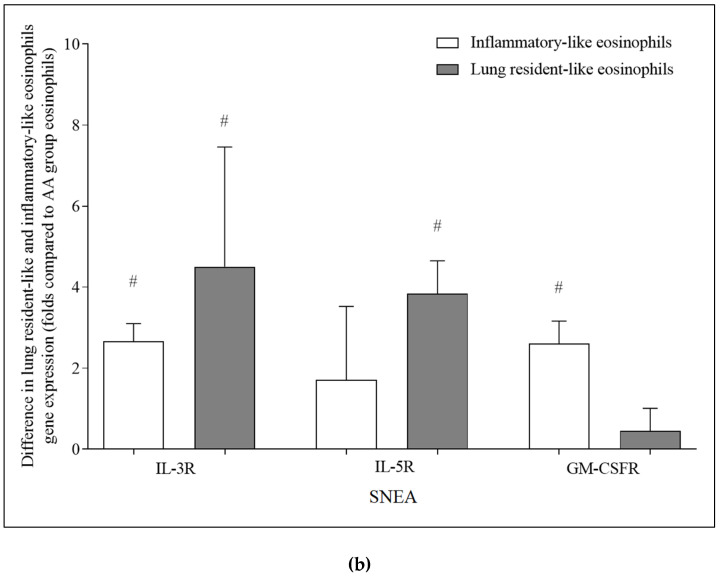
Differences in expression of IL-3R, IL-5R, and GM-CSFR genes between eosinophil subtypes. Comparison of (**a**) SNEA and AA groups with HS group; (**b**) SNEA group with AA group. IL-3R—interleukin 3 receptor; IL-5R—interleukin 5 receptor; GM-CSFR—granulocyte-macrophage colony-stimulating factor receptor. Severe nonallergic eosinophilic asthma (SNEA) group n = 10, nonsevere allergic asthma (AA) group n = 8, healthy subjects (HS) group n = 11. Data presented as the mean ± standard error of the mean, fold change over expression of IL-3R, IL-5R, and GM-CSFR gene in HS and AA groups. * *p* < 0.05 compared with HS group, ^#^ *p* < 0.05 compared with AA group. Statistical analysis: between investigated groups, Mann–Whitney two-sided U-test; within one study group, Wilcoxon matched-pair signed-rank two-sided test. Data presented as the mean ± standard error of the mean.

A higher expression of the IL-3 receptor gene was observed in iEOS-like and rEOS-like cells isolated from the SNEA group than from the HS group (2.1 ± 0.3- and 4.6 ± 2.6-fold, respectively, *p* < 0.05). A higher expression of the IL-5 receptor gene was observed in the iEOS-like and rEOS-like cells isolated from the SNEA and AA groups than in the iEOS-like cells isolated from the HS group (4.5 ± 2.6-, 4.3 ± 0.8-, 2.7 ± 0.9-, and 4.4 ± 1.7-fold, respectively, *p* < 0.05). A higher expression of the GM-CSF receptor gene was observed in rEOS-like cells isolated from the HS group than from the SNEA group (1.7 ± 0.6-fold, *p* < 0.05). Meanwhile, a higher expression of the GM-CSF receptor gene was observed in iEOS-like and rEOS-like cells isolated from the HS group than from the SNEA group (1.9 ± 0.9- and 2.4 ± 1.1-fold, respectively, *p* < 0.05) (Figure 5a).

A higher expression of the IL-3 receptor gene was observed in iEOS-like and rEOS-like cells isolated from the SNEA group than from the AA group (2.6 ± 0.4- and 4.5 ± 2.9-fold, respectively, *p* < 0.05). Meanwhile, a higher expression of the IL-5 receptor gene was observed in the rEOS-like cells isolated from the SNEA group than from the AA group (3.8 ± 0.5-fold, *p* < 0.05). A higher expression of the GM-CSF receptor gene was observed in iEOS-like cells isolated from the SNEA group than from the AA group (3.6 ± 0.5-fold, *p* < 0.05) (Figure 5b).

### 3.5. Effect of Allergen Activation on Expression of IL-3R, IL-5R, and GM-CSFR Genes

The effect on expression of eosinophilopoetin receptors of an in vivo provoked acute allergic asthma episode after bronchial allergen challenge was determined by comparing the results before and 24 h after the bronchial allergen challenge of the same subject.

**Figure 6:**
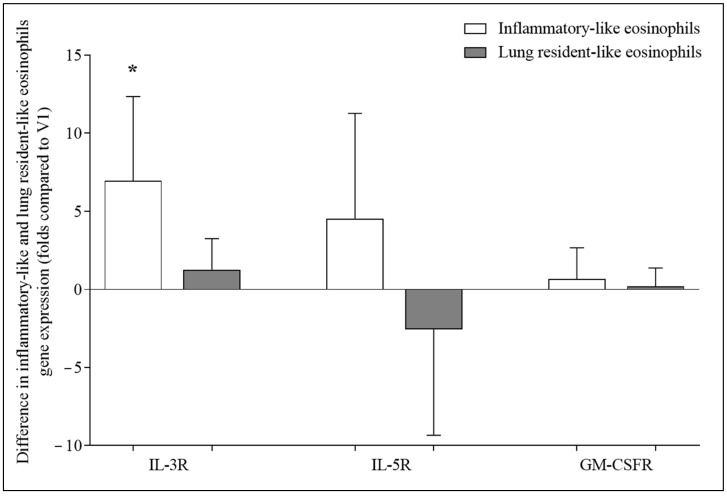
Differences in the expression of IL-3R, IL-5R, and GM-CSFR genes before and after bronchial allergen challenge. V2, visit 2 (24 h after bronchial allergen challenge). IL-3R—interleukin 3 receptor; IL-5R—interleukin 5 receptor; GM-CSFR—granulocyte-macrophage colony-stimulating factor receptor. Results from independent experiments of AA, n = 8. Data presented as the mean ± standard error of the mean, fold change over expression of IL-3R, IL-5R, and GM-CSFR gene after bronchial allergen challenge. * *p* < 0.05 compared with V2. Statistical analysis: between investigated groups, Mann–Whitney two-sided U-test; within one study group, Wilcoxon matched-pair signed-rank two-sided test. Data presented as the mean ± standard error of the mean.

Bronchial allergen challenge significantly increased the expression of the IL-3 receptor genes on iEOS-like cells compared with the same cells before the bronchial allergen challenge (7.0 ± 5.4 folds, *p* < 0.05). Meanwhile, the expression of the IL-5 and GM-CSF receptor genes did not significantly differ for both eosinophil subtypes before and after the bronchial allergen challenge (*p* > 0.05). (Figure 6).

## 4. Discussion

Eosinophilic inflammation predominates in half of asthmatic patients, and eosinophil counts in the airway and blood are associated with the severity of the disease. Eosinophils themselves as well as their maturation and activity in regulating cytokines called eosinophilopoetins are all prominent components of eosinophilic inflammation [6]. Our study results revealed that IL-5 and GM-CSF, but not IL-3, exhibited a significant effect on the proliferative properties of eosinophil subtypes in asthma patients. Moreover, we found that after the in vivo activation of AA patients by a bronchial allergen challenge, only rEOS-like cells demonstrated a tendency to be more activated by IL-5 and GM-CSF, with no effect on iEOS-like cells. Furthermore, eosinophil subtypes differed in the expression of eosinophilopoetin receptors; however, only IL-5R might be related with an effect on the proliferative properties of eosinophil subtypes.

Eosinophil is an active inflammatory cell that produces diverse cytokines, chemokines, and growth factors. During chronic airway inflammatory diseases, such as asthma, eosinophil activity is triggered, leading to abnormal infiltration into the airways and the disturbance of local homeostasis. At least two subtypes of eosinophils have been identified, and they differ according to their role in asthma pathogenesis. Studies identifying these populations in mouse models suggest that rEOS and iEOS have distinct biological roles: rEOS are homeostatic cells with a primary function of maintaining tissue homeostasis, while iEOS are mainly involved in immune responses [23]. This allows us to speculate that their effect on the physiology of lung structural cells, such as proliferation, also differs, which may be related to different responses to elevated levels of proinflammatory cytokines in patient blood. Our study is based on the blood-circulating eosinophils. These cells are released into the bloodstream in an active form and express airway tissue eosinophil-specific markers. For this reason, blood-circulating eosinophils could give sufficient information about their behavior before infiltration into asthmatic lungs.

Eosinophil development and functions depend on several cytokines, including IL-3, IL-5, and GM-CSF [24]. IL-3 and IL-5 are expressed mainly by activated T lymphocytes and mast cells. GM-CSF is produced by T cells, epithelial cells, and macrophages [10]. All three cytokines have a common beta (β)-chain receptor subunit and different specificities for alpha (α)-chain subunits. These three cytokines can differentially affect eosinophil functions due to the regulation and trafficking of their specific α-chain receptors on the eosinophil surface, and their specific downstream intracellular signaling in a βc-chain-independent manner [14].

Tai et al. demonstrated that any of the βc family cytokines increase blood eosinophil survival by preventing programmed cell death in human blood eosinophils [25]. Esnault et al. compared the effects of β-chain cytokines on eosinophil biology and demonstrated that IL-3 has a stronger effect than any other βc family cytokines in activating eosinophils [14]. However, despite the strong effect of IL-3 on eosinophil activity, the effects might be related to the stronger affinity to eosinophil IL-3 receptors rather than concentration in asthmatic patients’ blood [22].

The role of eosinophils in the development of airway remodeling, including the proliferation of ASM cells, was established relatively early [26]. ASM cells control muscle tone and thus regulate the opening of the airway lumen and air passage. Evidence indicates that ASM cells participate in airway hyperresponsiveness and the inflammatory and remodeling processes observed in asthmatic subjects [27]. The contribution of eosinophils to airway remodeling in asthma depends not only on their increased infiltration but also their survivability in airways, which prolongs their effect on pulmonary structural cells [8]. Previously, we demonstrated that the inclusion of either eosinophil subtypes in a coculture significantly increased the proliferation of ASM cells; however, no studies have been conducted with eosinophilopoetin-activated eosinophil subtypes to date. In this study, we aimed to determine the effect of eosinophils on the proliferative properties of eosinophil subtypes. Our use of combined eosinophilopoetin-activated blood eosinophil subtypes and the ASM cell culture model by simulating the processes in vivo revealed that IL-5 and GM-CSF, but not IL-3, are important for their proliferative properties. Both these cytokines significantly enhanced the effect of both iEOS-like and rEOS-like cells on ASM cell proliferation in AA and SNEA groups. Moreover, it was disclosed that activation by IL-5 and GM-CSF was more pronounced for the eosinophils of asthma patients than for the control HS. Ryan and colleagues found that IL-3 is the cytokine among the β-chain receptor cytokines that produces the most distinct eosinophil gene expression program; from our data, however, we can conclude that this dysregulation in the expression of this gene is not associated with the pro-proliferative properties of eosinophils.

People with asthma experience shortness of breath, cough, wheezing, and chest tightness. All these symptoms are associated with airway inflammation, obstruction, and airway remodeling [17]. This established correlation has prompted the development of eosinophil-targeting immunotherapies for decreasing eosinophilic count in the treatment of severe eosinophilic asthma. However, despite the biologic therapies, a subset of patients with severe eosinophilic asthma failed to achieve effective disease control [28]. The effect of IL-5 on the pro-proliferative properties of rEOS-like and iEOS-like cells might potentially be managed by anti-IL-5 antibodies; however, the condition remains poorly controlled by this treatment for some patients. A similar pro-proliferative effect of GM-CSF might be a suggestion for advanced and combined therapies targeting the main, eosinophils-related ASM remodeling in asthma. Moreover, the severe course of the disease, related to the group of SNEA patients with less control, indicates the enhanced response of iEOS-like cells to IL-5 and of rEOS-like cells to GM-CSF. This allows us to separate the responses between eosinophil subtypes and distinct eosinophilopoetins that, in combination with additional individual blood eosinophil subtypes-like cells quantity determination, could allow for the application of personalized treatment according to the disease phenotype.

The airways are constantly in contact with irritants, viruses, microbes, and allergens that can cause and exacerbate asthma [17]. During airway inflammation, when the airways are exposed to allergens, epithelial cells release signaling molecules that activate T helper 2 cells (Th2) or innate lymphoid type-2 cells (ILC2), which produce airway inflammatory cytokines [29,30]. To evaluate the effect of eosinophilopoetins in mimicking asthma exacerbations, we used the most common *D. pteronyssinus* house dust mite allergen with which humans constantly come into contact. iEOS-like cells are proinflammatory effector cells that are closely linked with immune responses. During a bronchial allergen challenge, these cells might be more strongly activated compared with resident eosinophils. However, our study revealed that in vivo activation after a bronchial allergen challenge enhances the proliferative properties of iEOS-like cells but does not increase their response to activation by any eosinophilopoetin. We can assume that iEOS-like cells are released in bone marrow in a more activated state or have a larger quantity of activated eosinophilopoetin receptors. Therefore, their pro-proliferative properties are less affected by increased levels of β-chain cytokines. However, Mesnil et al. (2016) described a distinct subtype of the eosinophil’s population, called rEOS, in inflammatory conditions [5]. This allows us to assume that steady-state rEOS-like cells might be less activated, and the bronchial allergen challenge, related to an acute asthma episode and a release of various proinflammatory cytokines, enhanced their response to IL-5 or GM-CSF and proliferative effects on ASM cells. We can speculate that enhanced ASM remodeling in acute asthma might be related to switched homeostatic rEOS-like cell functions and may be related to activation by IL-5 and GM-CSF but not to the more pronounced proliferative properties of iEOS-like cells.

Eosinophil activation by IL-3, IL-5, or GM-CSF depends not only on the concentration but on these cytokines and the number of β-chain cytokine receptors on the eosinophil surface. IL-3, IL-5, and GM-CSF share a common β-chain and have cytokine-specific α-chains [7]. Despite all three cytokines sharing a standard β-chain receptor subunit, each differentially affects the eosinophil biology due to the alpha (α) chain subunit-specific properties [31]. We assumed that the expression of the eosinophilopoetin receptor gene in eosinophil subtypes could play an important role in regulating the eosinophil proliferative properties, ASM cell proliferation, and airway structural changes. We sought to determine the expression of IL-3, IL-5, and GM-CSF receptor genes by focusing on expected eosinophilopoetin receptor counts on the eosinophil surface in different subject groups. Important differences between iEOS-like and rEOS-like cells were obtained regarding the expression of eosinophilopoetin receptor genes. rEOS-like cells were distinguished by a higher expression of the IL-3 receptor than in iEOS-like cells in both asthma groups. The IL-3 receptor gene expression of both eosinophil subtypes was significantly higher in the SNEA group than in the AA and HS groups; however, we found no relation between IL-3 activation and the proliferative properties of eosinophil subtypes. Thus, an increase in IL-3 receptors could be related to the biological properties of other eosinophil subtypes, especially for iEOS-like cells during acute asthma episodes, which needs to be investigated. Evaluating the expression of the IL-5 receptor gene, we found strong upregulation in both eosinophil-like cell subtypes of asthma groups compared with the HS group. We can conclude that increased IL-5R gene expression might be related to the enhanced proliferative properties of iEOS-like and rEOS-like cells after activation by IL-5.

GM-CSF receptor gene expression, as well as the IL-3 receptor, might not be related to the proliferative properties of eosinophil-like cell subtypes. Differently to IL-3R and IL-5R, the expression of the GM-CSFR gene of both subtypes in AA and in rEOS-like cells of SNEA patients was significantly downregulated compared with the HS group. β-Chain cytokine receptors have a specific ligand-related cross-regulation mechanism. Whole eosinophilopoetins can downregulate IL-5 receptor and upregulate IL-3 receptor expression, while GM-CSF receptors are downregulated by GM-CSF itself in rEOS under inflammatory conditions. Blood-circulating IL-5 and especially GM-CSF levels are enhanced in asthma [21]; therefore, it might be related to significantly lower GM-CSFR mRNR levels in blood eosinophil subtype-like cells in asthma and less activation of eosinophil by GM-CSF in our in vitro model. However, the quantity of eosinophilopoetin receptors is not the main reason for signal transduction. The receptors of eosinophilopoetin exist as monomers on unstimulated cells—these are inactive, while signal transmission is possible only after activation. Eosinophilopoetins bind to their respective α-chain with low affinity, and the subsequent recruitment of the β-chain contributes to a conformational change with a significant association with binding in a complex. The enhancement of the proliferative properties of eosinophil subtypes after activation with GM-CSF might be due to more receptors being in an activated state. Moreover, iEOS-like cells of SNEA patients could be distinguished from the population of rEOS-like cells by enhanced GM-CSF receptor gene expression. This difference could be considered a characteristic feature of SNEA, which allowed us to predict the GM-CSF cytokine as a possible target to reduce the effect of iEOS-like cells on ASM remodeling.

We established that the effect of IL-5 and GM-CSF on the eosinophil subtypes significantly increased the ASM cell number. It leads to the thickening of the airway wall and plays an important role in airway remodeling. For this reason, ASM cell proliferation can be associated with a severe course of asthma and weakened lung function. The United States Food and Drug Administration (FDA) approved the first humanized monoclonal antibody, which blocks IL-5 (mepolizumab), to treat the severe asthma eosinophilic phenotype in 2015. After 2 years, the same institution approved the second humanized monoclonal antibody, which blocks IL-5 receptors (benralizumab). Anti-IL-5 and anti-IL-5 receptor antibody treatment significantly decrease the frequency of asthma exacerbations and the use of systemic glucocorticoids, improving lung function and quality of life [31,32,33,34,35].

Based on the data, we think that the GM-CSF block might be an alternative target in treating asthma. The first study in asthma patients with an anti-GM-CSF monoclonal antibody was performed in 2012–2014. This two-phase study’s main aim to improve lung function was not met in the overall population; however, forced expiratory volume in 1 s (FEV1) significantly increased in the group of patients with >300 peripheral blood eosinophils/mL at baseline. No significant effect in reducing asthma exacerbations or improving asthma control was found in this study as well. It is being considered that the study population or study medication dose was not a good choice [36]. To date, no further clinical trials have been conducted with anti-GM-CSF antibodies.

Our study had several limitations. A potential restriction of the eosinophil subtype model used in the study is that after the isolation of the total blood eosinophil population from granulocytes, rEOS-like cells were isolated by positive selection against CD62L, keeping iEOS-like cells as CD62L negative cells. However, the isolated eosinophil population was not 100% pure, and other CD62-L negative blood cells can contaminate the population of iEOS-like cells. Incidentally, not all the purified eosinophils remained viable after 72 h of incubation with ASM cells. Therefore, the proliferation data in this study may be related to residual viable eosinophils. However, we assessed that activated eosinophils can rapidly degranulate and release proliferative mediators that affect ASM cells even when eosinophils are nonviable. Moreover, in the AA group, eosinophils were assessed during the active allergic reaction after the bronchial allergen challenge but not during the late allergic reaction. We think eosinophils may have been more activated in the late allergic reaction due to more pronounced eosinophilic inflammation. We investigated the expression levels of IL-3, IL-5, and GM-CSF receptor genes. As gene expression does not always reflect the levels of maturate receptors, our conclusions based only on the expression of eosinophilopoetin receptor genes is limited.

In conclusion, IL-5 and GM-CSF demonstrated a significant effect on the proliferative properties of eosinophil subtypes in patients with asthma; the exposure of these eosinophilopoetins to eosinophil subtypes significantly increased the ASM cell number. However, ASM cell proliferation was associated with the expression of only the IL-5 receptor gene in eosinophil subtypes. Though we did not detect an IL-3 effect on eosinophil proliferative properties, IL-3 undoubtedly acts on the eosinophil by altering its other functions, but more research is needed to determine the details.

## Figures and Tables

**Table 1:** Inclusion and exclusion criteria.

	SNEA Group	AA Group	HS
Inclusion criteria	Asthma history ≥ 12 months Negative skin prick test Peripheral blood eosinophil ≥ 0.3 × 10^9^/L High doses of inhaled steroids + long-acting beta agonist + episodic use of oral steroids	Asthma symptoms ≥ 12 months Nonsevere course of the disease Inhaled steroids free period of at least 3 months Positive skin prick test to *D. pteronyssinus* Positive metacholine challenge test/positive bronchial reversibility test	No chronic respiratory or other lung diseases Negative skin prick test
Exclusion criteria	Clinically significant allergy symptoms Active airway infection ≤ 1 months prior to study Coronavirus infectious disease 2019 (COVID-19) ≤ 1 months prior to study Asthma exacerbation ≤ 1 months prior to study Use of oral steroids ≤ 1 months prior to study Active smoking (at least one cigarette a day) Former smoker (at least 100 cigarettes in lifetime)		

AA—allergic asthma; HS—healthy subjects; SNEA—severe nonallergic eosinophilic asthma.

**Table 2:** Research plan.

	SNEA Group (n = 18)	AA Group (n = 17)	HS (n = 11)
Screening visit (visit 1)			
Inclusion and exclusion criteria	√	√	√
Written informed consent	√	√	√
CBC	√	√	√
Spirometry	√	√	√
Metacholine challenge test/bronchial reversibility test	ND	√	ND
Skin prick test	√	√	√
Experimental day (visit 2)			
CBC	√	√	√
IgE	√	√	√
Fe_NO_ measurement	√	√	√
Spirometry	√	√	√
Bronchial challenge with *D. pteronyssinus*	ND	√	ND
Experimental day 24 h after bronchial allergen challenge (visit 3)			
CBC	ND	√	ND
IgE	ND	√	ND
Fe_NO_ measurement	ND	√	ND
Spirometry	ND	√	ND

AA—allergic asthma; CBC—complete blood count; Fe_NO_—fractional exhaled nitric oxide; HS—healthy subjects; IgE—immunoglobulin E; ND—not done; SNEA—severe nonallergic eosinophilic asthma.

**Table 3:** Demographic and clinical characteristics of the study population.

	SNEA Patients	AA Patients	HS
Number, n	18	17	11
Sex, M/F	4/14	12/5	3/8
Age, years	57.8 ± 2.4 *^#^	28.1 ± 2.4	31.4 ± 3.2
BMI, kg/m^2^	29.9 ± 1.6 *^#^	24.0 ± 0.9	25.3 ± 1.2
FEV_1_, L	1.6 ± 0.1 *^#^	3.7 ± 0.2	3.7 ± 0.2
FEV_1_, % of predicted	58.9 ± 4.7 *^#^	86.8 ± 2.4 ^#^	99.1 ± 3.5
PD_20M_, mean (range), mg	ND	0.22 ± 0.34	ND
PD_20A_, mean (range), HEP/mL	ND	14.12 ± 1.92	ND
Blood eosinophil count, ×10^9^/L	0.63 ± 0.09 *^#^	0.39 ± 0.05 ^#§^	0.17 ± 0.02
Blood eosinophil count 24 h after allergen challenge, ×10^9^/L	ND	0.51 ± 0.06	ND
Fe_NO_, ppb	49.1 ± 8.3 ^#^	51.6 ± 8.9 ^#§^	10.8 ± 1.9
Fe_NO_, 24 h after allergen challenge, ppb	ND	78.2 ± 12.6	ND
IgE, IU/mL	158.8 ± 45.2 *^#^	837.3 ± 316.7 ^#^	18.2 ± 5.0
IgE, 24 h after allergen challenge, IU/mL	ND	861.1 ± 323.1	ND

AA—allergic asthma; BMI—body mass index; F—female; Fe_NO_—fractional exhaled nitric oxide; FEV_1_—forced expiratory volume in 1s; IgE—immunoglobulin E; M—male; ND—not done; PD_20A_—the *D. pteronyssinus* allergen provocation dose causing a 20% decrease in FEV_1_; PD_20M_—the provocation dose of methacholine causing a 20% decrease in FEV_1_; SNEA—severe nonallergic eosinophilic asthma. Data presented as mean ± standard error of the mean. * *p* < 0.05 compared with AA group. ^#^ *p* < 0.05 compared with HS group. ^§^ *p* < 0.05 compared with AA group after allergen challenge.

## Data Availability

All the data presented in this study are included in this article.
